# Once again on rapamycin-induced insulin resistance and longevity: despite of or owing to

**DOI:** 10.18632/aging.100461

**Published:** 2012-05-31

**Authors:** Mikhail V. Blagosklonny

**Affiliations:** Department of Cell Stress Biology, Roswell Park Cancer Institute, Buffalo, NY, USA

**Keywords:** aging, diabetes, retinopathy, nephropathy, diseases, anti-aging drugs, growth hormone

## Abstract

Calorie restriction (CR), which deactivates the nutrient-sensing mTOR pathway, slows down aging and prevents age-related diseases such as type II diabetes. Compared with CR, rapamycin more efficiently inhibits mTOR. Noteworthy, severe CR and starvation cause a reversible condition known as “starvation diabetes.” As was already discussed, chronic administration of rapamycin can cause a similar condition in some animal models. A recent paper published in Science reported that chronic treatment with rapamycin causes a diabetes-like condition in mice by indirectly inhibiting mTOR complex 2. Here I introduce the notion of benevolent diabetes and discuss whether starvation-like effects of chronic high dose treatment with rapamycin are an obstacle for its use as an anti-aging drug.

## Starvation diabetes-like condition with low mTOR activity

If you read the Abstract, you might wonder whether rapamycin extends lifespan despite or because of “starvation-like diabetes”. As described by Lamming et al [1, 2] extending several previous observations [3-6], chronic administration of high doses of rapamycin causes insulin resistance in mice. Yet, at similar doses, rapamycin prolongs life span in mice [7, 8]. Moreover, in several studies, rapamycin prevented complications of diabetes such as nephropathy [9-14]. Also, theoretical considerations indicate rapamycin for retinopathy [15], which was recently confirmed in an animal model [16]. Rapamycin prevents atherosclerosis in rodents [17-20] and coronary re-stenosis in humans [21, 22]. In contrast, diabetes promotes nephropathy, retinopathy, atherosclerosis and coronary disease. How could this be reconciled? mTOR is a part of a nutrient-sensing pathway [23-27]. Nutrients and insulin activate mTOR. Rapamycin, which inhibits mTOR, is a “starvation-mimetic”, making the organism “think” that food is in a short supply. The most starvation-sensitive organ is the brain. The brain consumes only glucose and ketones. Therefore, to feed the brain during starvation, the liver produces glucose from amino acids (gluconeogenesis) and ketones from fatty acids (ketogenesis). Since insulin blocks both processes, the liver needs to become resistant to insulin. Also secretion of insulin by beta-cells is decreased. And adipocytes release fatty acids (lipolysis) to fuel ketogenesis by the liver. Thus, there are five noticeable metabolic alterations of starvation: gluconeogenesis, ketogenesis, insulin resistance, low insulin levels and increased lipolysis. This metabolic switch is known as starvation diabetes, a reversible condition, described 160 years ago (see for references [28]). Starvation diabetes could be explained by deactivation of mTOR, which otherwise is activated by nutrients. In theory, rapamycin can cause similar symptoms in the presence of nutrients.

## Type II diabetes: insulin-resistance due to active mTOR

**Figure 1:**
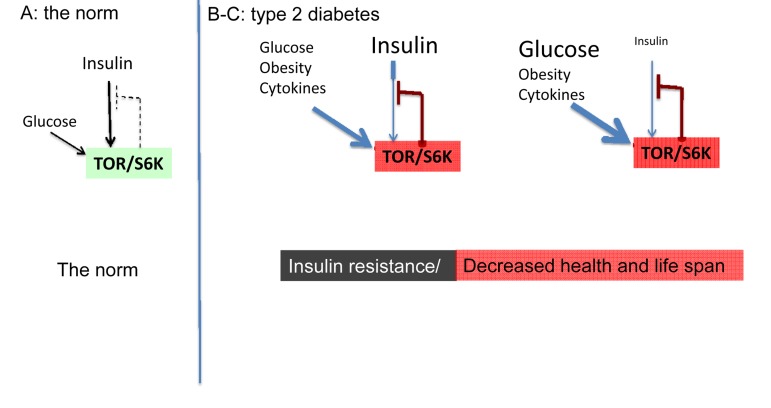
The norm and type 2 diabetes (simplified schema) (**A**) **The norm.** Insulin and nutrients such as glucose stimulate mTOR, which blocks insulin signaling (feedback loop). (**B-C**) **High mTOR/S6K activity: insulin resistance plus decreased lifespan.** (**B**) Overactivated by nutrients, cytokins, insulin and other hormones, mTOR blocks insulin signaling causing insulin resistance. Nutrients overstimulate beta-cells and insulin is increased. (**C**) In type II diabetes, beta-cells eventually fail and levels of insulin may be decreased.

Starvation-diabetes is not a true type II diabetes. Type II diabetes is a consequence of insulin-resistance in part due to excessive nutrients and obesity. Even brief overfeeding may induce insulin resistance [29]. Nutrients and insulin activate mTOR. In turn, over-activated mTOR causes insulin resistance [30-42]. This feedback loop is shown in figure 1A. mTOR activates S6 kinase (S6K), which causes degradation of insulin-receptor substrates (IRS), thus impairing insulin signaling. Also, mTOR causes insulin resistance by an additional feedback mechanism [43, 44].

In high fat-fed obese rats, the mTOR pathway is activated in the liver and muscle, leading to insulin resistance [35]. In mice, sustained activation (by high fat feeding) of mTOR is associated with hepatic insulin resistance [45]. Chronic increase of insulin levels (hyperinsulinemia) causes insulin resistance, preventable by rapamycin [46]. In some animal models, removal of visceral fat prevents insulin resistance [47-49]. In humans, infusion of amino acids activate mTOR/S6K1, causing insulin resistance [38, 40]. In healthy men, rapamycin prevented activation of mTOR and insulin resistance caused by amino acid mixture [50]. Insulin stimulates glucose uptake and also activates mTOR. By a feedback loop, mTORC1 promotes insulin-resistance, decreasing glucose uptake by the cell. And most detrimentally, mTOR is involved in diabetic complications and age-related diseases [24-27, 51-54].

## The two opposite conditions?

Type II diabetes and starvation diabetes seem to be the two opposite conditions: the first is associated with activation of nutrient-sensing pathways, whereas the second is associated with deactivation of nutrient sensing pathways such as mTOR. Type II diabetes is dangerous by its complications such as retinopathy, neuropathy and accelerated atherosclerosis and cancer. Long-term effects of prolonged “starvation diabetes” is not known of course: it could not last for a long time, otherwise an animal (or human) would die from starvation. Or would not? An outstanding study by Fontana et al provides some answers [55]. Among individuals who had been practicing sever CR for an average of 7 years, 40% of CR individuals exhibited “diabetic-like” glucose intolerance, despite low levels of fasting glucose, insulin and inflammatory cytokines as well as excellent other metabolic profiles. In comparison with the rest CR individuals, they had lower BMI, leptin, circulating IGF-I, testosterone, and high levels of adiponectin, which are key adoptations to CR in rodents, suggesting severe CR [55]. The authors speculated that the “insulin resistance” in this severe CR group might have the effect of slowing aging, also based on the finding that a number of insulin-resistant strains of mice are long-lived [55]. The same conclusion could be reached from the mTOR perspective (Appendix 1).

“The paradox of the insu-lin/IGF-1 signaling pathway in longevity” was first discussed by Nir Barzilai and co-workers, who precisely noticed that insulin-resistance, which is so detrimental in obese and aging mammals, can be associated with genetic manipulations that extend life span in model organisms [56]. Later Barzilai et al suggested that insulin-resistance might serve as an adaptive mechanism in some tissues by preventing excess uptake of nutrients by cells [57]. This very interesting idea implies that insulin resistance is partially beneficial and partially hazardous in the same condition such as type II diabetes. But still insulin resistance in type II diabetes is overall harmful (leading to retinopathy and other complications), whereas insulin resistance during severe CR is benevolent. These are clearly different conditions. In fact, they are the opposite conditions. So insulin resistance may be harmful or beneficial depending on the underlying condition.

**Figure 2:**
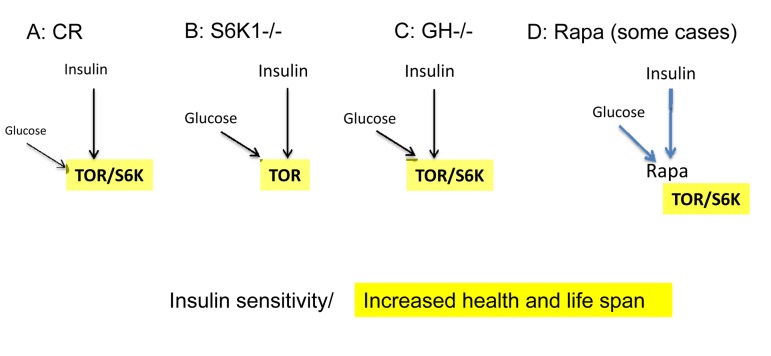
Low mTOR/S6K activity: insulin sensitivity plus longevity (**A**) **Calorie restriction.** Deactivation of the nutrient-sensing mTOR pathway results in insulin sensitivity. (**B**) **Knockout of S6K1** in mice abolishes feedback block of insulin signaling, resulting in insulin sensitivity [94]. (**C**) **Decreased levels of growth hormone (GH).** In mice, absence of GH or GH receptor leads to a remarkable extension of longevity [95]. GH receptor deficiency is associated with a reduction in pro-aging signaling, cancer, and diabetes in humans [96]. Growth hormone signaling accelerates aging in mammals [97]. Remarkably, growth stimulation promotes cellular aging, when cells cannot proliferate [98, 99]. Thus, the growth promoting pathways such as mTOR are involved in both organismal and cellular aging. (**D**) **Acute treatment with rapamycin.** Deactivation of the nutrient-sensing mTOR pathway abolishes a feedback block of insulin signaling, resulting in insulin sensitivity [50].

**Figure 3:**
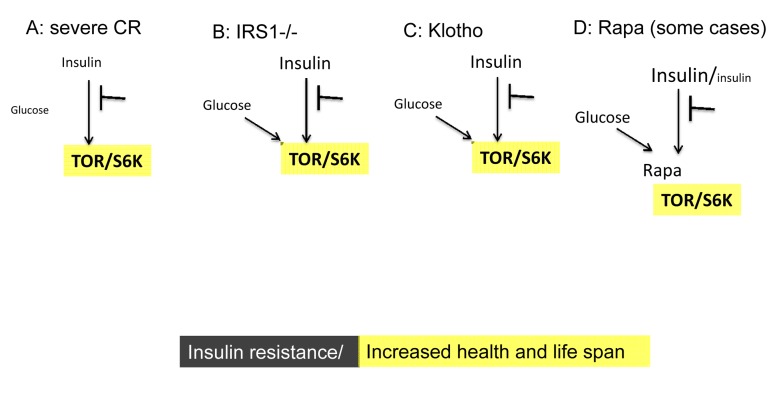
Low TOR/S6K activity: insulin resistance plus longevity (type 0 diabetes) (**A**) **Severe CR and starvation.** Insulin resistance and symptoms of diabetes are observed during starvation [28] and prolong severe CR [55]. Furthermore, CR may reduce rather than enhance insulin effects in the insulin-sensitive dwarf mice [100]. (**B**) **IRS1 knockout.** Insulin receptor substrate 1 null mice live longer despite insulin resistance [101]. (**C**) **Klotho mice.** Overexpression of Klotho in mice extends life span. Klotho protein represses intracellular signals of insulin and insulin-like growth factor 1 (IGF1), [102]. Also, Klotho interferes with insulin/IGF-like signaling to improve longevity in *Caenorhabditis elegans* [103]. (**D**) **Chronic treatment with high doses of rapamycin** causes insulin resistance and glucose intolerance. This condition can be associated with normal/increased and decreased levels of insulin. Noteworthy, rapamycin induces Klotho [64].

The model of TOR-driven hyper-functional aging almost automatically solves paradoxes of aging, including the insulin paradox (see paradox 7 and figure 4 in “Paradoxes of aging” [58]). From the TOR perspective, insulin resistance is beneficial or harmful when it is associated with ether low or high TOR activity, respectively (Appendix, Fig. 1 and 3). And this should not be surprising. Consider insulin resistance as a symptom. The assessment of symptoms depends on the underlying cause. For example, weight loss due to calorie restriction is good, whereas weight loss in terminal cancer is bad. Positive Tuberculosis Skin (PPD) Test due to vaccination indicates protection from tuberculosis, whereas positive test due to tuberculosis is a symptom of tuberculosis. Similarly, hyperlipidemia in obesity is bad, whereas hyperlipidemia due to rapamycin-induced lipolysis is good (see figure 2 in reference [53]). The list of examples is endless. Similarly, insulin resistance, associated with TOR overactivation, is bad (Fig. 1 B-C). But either insulin sensitivity (Fig. 2) or insulin resistance (Fig. 3), associated with inactive TOR, is good.

## Type zero or benevolent diabetes

There are two types of diabetes, which at advanced stages may become similar. Insulin resistance may develop in type I diabetes (due to high glucose), whereas insulin insufficiency in type II diabetes (due to loss of beta-cells). Both types of diabetes lead to complications. In comparison, starvation diabetes [28] is only superficially resembles either type of diabetes. Also, diabetes-like symptoms may occur in rapamycin-treated mice and animals with genetically inhibited insulin/IGFI signaling (Fig. 3). To encompass all these cases, I suggest the term type 0 (zero) or benevolent diabetes. It is possible that some patients with diabetes have inactivating mutations in the insulin/IGFI pathway and thus “suffer” from benevolent diabetes. Furthermore, the condition can be imitated by chronic administration of rapamycin at least in some strains of mice. Both calorie restriction and rapamycin extend life span in mice. Rapamycin prevents retinopathy and nephropathy. Also CR prevents type II diabetes and other diseases [59], [60], [61], [62]. One can suggest that type 0 diabetes should prevent type 2 diabetes. Should type 0 diabetes be treated? Perhaps CR-associated type 0 diabetes should not. What about rapamycin-associated diabetes? Definitely, it should not be treated with insulin. It was discussed that in theory the most rational combinations with rapamycin are mild calorie and fat restriction, physical exercise and metformin [52]. Metformin may in theory counteract rapamycin-induced gluconeogenesis in the liver. And this rational drug combination may be also considered as treatment of type 0 diabetes.

## Inconsistencies in the literature on rapamycin-induced insulin resistance

As demonstrated by Lamming et al, chronic administration of rapamycin caused insulin-resistance due to deactivation of mTORC2 and Akt [1]. This is consistent with previous data that IRS signaling and AKT activation was impaired in patients treated with rapamycin [63]. However, there are some inconsis-tencies. In another clinical study, rapamycin therapy in contrast caused activation of Akt [64]. Second, whereas Lamming et al found that rapamycin increased insulin levels after feeding [1], other studies reported that rapamycin in contrast inhibited insulin secretion [3, 4, 65]. Furthermore, inhibition of beta-cell adaptation and insulin production by rapamycin was considered as the main mechanism of rapamycin-induced diabetes in mice [6, 66-69]. On the other hand, selective inactivation of mTORC2 in the liver can cause hyperinsulinemia [70].

Finally, diabetic-like symptoms were not observed in numerous studies in mice. And rapamycin-induced diabetes is rare in human patients, even though most of them are prone to diabetes for other reasons.

## Diabetes in patients receiving rapamycin

In renal transplant patients, who are prone to diabetes (due to several reasons), chronic administration of rapamycin modestly increases incidence of diabetes [71, 72]. Although the increase is statistically significant, it took many years to detect it. For many years it was thought that, unlike other agents used in these patients, rapamycin either do not increase the incidence of diabetes or increases it in combinations with tacrolimus [73-79]. In the study involving 20124 recipients of kidney transplant sirolimus (rapamycin) was independently associated with new onset diabetes [72]. And although it statistically significantly increases the incidence of diabetes in renal transplant patient, we do not know whether this is true diabetes, which is dangerous by its complications, or starvation-like diabetes, that prevents the complications of true diabetes . Will chronic high doses of rapamycin cause or prevent diabetes in humans without organ transplantation? More investigations are needed.

## Intermittent administration of rapamycin

Is glucose intolerance a part of therapeutic effects of starvation-like drugs such as rapamycin? And may such condition be not only benign but also prevent true diabetes and its complications? Although these questions are very intriguing, the answers are not immediately crucial. Simply, the most rational anti-aging schedule is an intermittent (rather than chronic) administration of rapamycin [53, 80]. First, this will eliminate potential side effects. Second, intermittent administration of rapamycin may in theory rejuvenate stem and wound-healing cells and (in contrast to chronic treatment) improve wound healing [80]. And intermittent administration of rapamycin extended life span in mice [81-86]. Also, brief treatment with rapamycin does not affect mTORC2 [87].

Rapalogs (rapamycin and its analogs such evirolimus and temsirolimus) inhibit only one target (mTORC1). That was considered as a disadvantage of rapalogs for cancer therapy. Inhibitors of both mTORC1 and mTORC2 are under development [88, 89]. But if inhibition of mTORC2 is not needed for the longevity effect, then mTORC1 selectivity is an advantage for anti-aging therapy. Rapalogs (rapamycin and its analogs) are selective inhibitors of TORC1 and inhibitors of mTORC1 will have the same side effects as rapalogs. Yet, these (non-rapalog) inhibitors of the TOR kinase also have off-target effects and side effects. Therefore, rapamycin will remain the least toxic anti-aging drug in the near future [90].

## Appendix 1: Paradoxes of diabetes

Previously I discussed that the mTOR-centered model can solve so called insulin paradox [58], which was exploited by Tom Kirkwood to undermine the notion that aging is genetically regulated: “it seems paradoxical that reduced insulin/ IGF-1 signaling extends life span but insulin resistance leads to type II diabetes. The real paradox is why, in mammals, low insulin levels are associated with good health, but low insulin responsiveness with bad health” [91]. In other words, (a) low insulin signaling are associated with good health and longevity and (b) insulin resistance is associated with poor health. In both cases (A and B), the insulin signaling is decreased. So why? From the mTOR point of view, the cases are opposites. In case A (Fig. 2), low insulin signaling is insufficient to activate mTOR (and this is good). In case B (Fig. 1 B-C), insulin signaling is low because of the active mTOR (this is bad), which blocks insulin signaling. In case B, active mTOR is a cause of insulin resistance and low insulin signaling (Fig. 1 B-C). In case A, low insulin signaling keeps mTOR inactive (Fig. 2).

Noteworthy, dwarf (GH-/-) (Fig. 2) and Klotho (Fig. 3) mice have an extended longevity. But Klotho induces IGF-1 and insulin resistance, whereas dwarf mice with reduced IGF-1 and insulin levels have enhanced insulin sensitivity. Bartke et al suggested that signaling downstream from IGF-1 and insulin receptors is reduced in both Klotho and dwarf mice [92, 93]. This is in agreement with the mTOR-centric model [58], given that the mTOR pathway is downstream from insulin/IGF receptors.

The same mTOR-centered point of view is applicable to the diabetes paradox. When diabetes is caused by high mTOR activity, then it is associated with complications, diseases and shortened life span (Fig. 1). This is type 2 diabetes. But when diabetic-like condition is caused by either starvation or rapamycin, then it might be benevolent (Fig. 3).
